# Anticlusterin treatment of breast cancer cells increases the sensitivities of chemotherapy and tamoxifen and counteracts the inhibitory action of dexamethasone on chemotherapy-induced cytotoxicity

**DOI:** 10.1186/bcr1835

**Published:** 2007-12-13

**Authors:** Maximino Redondo, Teresa Téllez, Maria J Roldan, Alfonso Serrano, Maria García-Aranda, Martin E Gleave, Maria L Hortas, Miguel Morell

**Affiliations:** 1 Department of Biochemistry, Hospital Costa del Sol, Carretera de Cádiz Km 187, 29600 Marbella, Málaga, Spain; 2 Department of Biochemistry and Immunology, Virgen de la Victoria University Hospital, Campus Universitario Teatinos, 29010 Málaga, Spain; 3 Prostate Center at Vancouver General Hospital, 2660 Oak Street, Vancouver, BC V5Z 3J5, Canada

## Abstract

**Introduction:**

Overexpression of the apoptosis-related protein clusterin is associated with breast cancer development and tumor progression. We describe the use of clusterin-specific antisense oligonucleotides and antibodies to sensitize breast carcinoma cells to anticancer drugs routinely used in breast cancer therapy.

**Methods:**

MCF-7 and MDA-MB-231 cells were treated with the oligonucleotide or antibody, chemotherapeutic agents (doxorubicin or paclitaxel), tamoxifen, or with combinations of these.

**Results:**

Treatments that include antisense clusterin oligonucleotide or antibody to clusterin have been shown to reduce the number of viable cells more effectively than treatment with the drugs alone. We also demonstrate that dexamethasone pretreatment of breast cancer cell lines inhibits chemotherapy-induced cytotoxicity and is associated with the transcriptional induction of clusterin. However, anticlusterin treatment increases chemotherapy-induced cytotoxicity, even in the presence of glucocorticoids, suggesting a possible role for these proteins in glucocorticoid-mediated survival.

**Conclusion:**

These data suggest that combined treatment with antibodies to clusterin or antisense clusterin oligodeoxynucleotides and paclitaxel, doxorubicin, or tamoxifen could be a novel and attractive strategy to inhibit the progression of breast carcinoma by regulation of the clusterin function. Moreover, glucocorticoid activation in breast cancer cells regulates survival signaling by the direct transactivation of genes like clusterin which encode proteins that decrease susceptibility to apoptosis. Given the widespread clinical administration of dexamethasone before chemotherapy, understanding glucocorticoid-induced survival mechanisms is essential for achieving optimal therapeutic responses.

## Introduction

Breast cancer is the most frequently diagnosed cancer in women today, and its incidence has steadily increased in recent decades. Resistance to anticancer chemotherapeutic drugs remains a major obstacle in cancer chemotherapy, and novel therapeutic strategies that target the molecular basis of chemoresistance are required. In this sense, the response of cytotoxic drugs is modulated by pro- and antiapoptotic proteins, and defects in apoptosis pathways or the activation of antiapoptotic mechanisms may confer resistance to cytotoxic drug treatment. In fact, the downregulation of the CD-95 receptor/ligand system, deficient expression of caspase family members, or the overexpression of antiapoptotic bcl-2 protein have all been observed in drug-resistant tumors [1]. The clusterin protein is an inhibitor of apoptosis with a cytoprotective function [2] and thus represents a promising target for molecular intervention strategies such as antisense therapy designed to inhibit its expression [3]. The overexpression of exogenous clusterin has been shown to result in resistance to paclitaxel [4], doxorubicin [5], cisplatin [6], and radiation therapy [7]. In contrast, decreased clusterin expression by antisense or small interfering RNA (siRNA) expression enhances the chemosensitivities of various cell lines [8-11], suggesting that clusterin expression is a prominent resistance factor in cancer cells.

On the other hand, glucocorticoids (such as dexamethasone) are routinely used in the clinical application of chemotherapy to prevent adverse effects. A previous study reported the inhibitory action of glucocorticoids on chemotherapy-induced apoptosis, which also raises a clinically relevant question as to whether the pretreatment with glucocorticoids might interfere with the therapeutic efficacy of chemotherapy [12].

Glucocorticoids play a major role in attenuation of the inflammatory response. These steroid hormones are able to induce apoptosis in cells of the hematopoietic system such as the monocytes, macrophages, and T lymphocytes that are involved in the inflammation reaction. In contrast, it has recently been discovered that in glandular cells such as the mammary gland epithelia, hepatocytes, and ovarian follicular cells and in fibroblasts, glucocorticoids protect against the apoptotic signals evoked by cytokines, cAMP, tumor suppressors, and death genes. It is well known that the antiapoptotic effect of glucocorticoids is exerted by the modulation of survival genes such as Bcl-2, Bcl-x(L), and nuclear factor-kappa B in a cell type-specific manner [13]. We hypothesize that clusterin may be one of these genes responsible for the antiapoptotic effect of glucocorticoids.

Increased expression of the clusterin gene has been observed in breast cancer cells and has been associated with the development and progression of these tumors [14]. Moreover, clusterin overexpression has been shown to be associated with the anti-HER-2 antibody trastuzumab (Herceptin) treatment resistance through the inhibition of apoptosis [15]. To explore the potential of the clusterin inhibition approach in breast cancer therapy, the cytotoxic interaction between antisense clusterin oligonucleotide or anticlusterin antibody and the drugs commonly used in breast cancer treatment such as dexamethasone, doxorubicin, paclitaxel, and tamoxifen were analyzed *in vitro *using the breast carcinoma cell lines MCF-7 and MDA-MB-231.

## Materials and methods

### Cell lines

Estrogen-independent MDA-MB-231 and estrogen-dependent MCF-7 breast cancer cell lines were obtained from the American Type Culture Collection (Manassas, VA, USA). They were maintained in RPMI 1640 (Sigma-Aldrich, St. Louis, MO, USA) supplemented with 10% fetal bovine serum.

### Anticancer drugs

Dexamethasone, tamoxifen, doxorubicin, and paclitaxel were obtained from Sigma-Aldrich. Stock solutions were prepared with phosphate-buffered saline to the required concentrations before each *in vitro *experiment.

### Total RNA isolation and reverse transcription-polymerase chain reaction

Isolation of total RNA was performed by the guanidium isothiocyanate method as originally described by Chomczynski and Sacchi [16]. In brief, 1 × 10^7 ^cells or 100-mg tissue fragments were first resuspended in 500 μL of a guanidium isothiocyanate solution. After phenol/chloroform extraction, 400 μL of the upper aquatic phase was transferred into a new tube, and RNA was precipitated with 2.5 volumes of absolute ethanol. The purity and integrity of the isolated RNA were determined by spectrophotometry and denaturing formaldehyde agarose gel electrophoresis, respectively. If necessary, isolated total RNA was treated with 2 units of RNase-free DNase I (Roche Molecular Biochemical, Mannheim, Germany) for 2 hours at 37°C to remove all possible genomic DNA contamination. Reverse transcription into cDNA was performed using a commercial kit (Roche Molecular Biochemical) in accordance with the manufacturer's instructions. Polymerase chain reaction (PCR) was performed using the following primers for detection of clusterin and α-actin signals, respectively: clusterin: 5'-GGCGACGATGACCGGACTGT-3' and 5'-GGGACCGTCACAGTGATGGG-3'; β-actin: 5'-GGCATCGTGATGGACTCCG-3' and 5'-GCTGGAAGGTGGACAGCGA-3'.

Using reverse-transcribed cDNA, all PCRs were performed in accordance with the following protocol: 5 minutes of denaturation at 95°C, 30 cycles of a 30-second denaturation step at 95°C, 1-minute annealing at 64°C, 30-second elongation at 72°C, and a final elongation step for 5 minutes at 72°C. PCR was performed on a GeneAmp 2400 thermal cycler (PerkinElmer Inc., Waltham, MA, USA) using 0.5 units of Taq polymerase (Roche) per reaction. The PCR products were analyzed in a 1.5% TAE (Tris Acetate EDTA)-buffered agarose gel.

### Changes in clusterin expression

To determine whether tamoxifen and dexamethasone altered clusterin expression, the cells were treated with charcoal-stripped serum (Merck & Co., Inc., Whitehouse Station, NJ, USA) to remove steroids. All drugs were assayed at concentrations of 10^-7 ^and 10^-8 ^M. Clusterin levels were determined at 4, 8, 24, 48, 96, 120, and 144 hours.

### Treatment of cells with oligonucleotides

The phosphorothioate oligodeoxynucleotides (ODNs) used in this study were provided by Roche Molecular Biochemical. The sequence of oligonucleotides to clusterin, corresponding to the mouse clusterin translation initiation site, was 5'-GCACAGCAGGAGAATCTTCAT-3'. A two-base clusterin mismatch ODN (5'-GCACAGCAGGAGGATATTCAT-3') was used as a control. These sequences of oligonucleotides have been previously shown to reduce levels of clusterin and to significantly delay disease progression in prostate and bladder tumor models in animals [8,17]. Furthermore, a second generation of this antisense oligonucleotide has been produced for use in humans (OGX-011, a 2' methoxyethyl modified phosphorothioate antisense oligonucleotide; OncoGenex Technologies Inc., Vancouver, BC, Canada).

*In vitro*-cultured cells were treated with various concentrations of ODN (ranging from 10 to 500 nM) after a preincubation for 20 minutes with 1.5 μg/mL lipofectin (Life Technologies, Glasgow, UK) to enhance transfection in serum-free Dulbecco's modified Eagle's medium (Sigma-Aldrich). Four hours after the beginning of the incubation, the medium containing ODN and lipofectin was replaced with standard medium. To determine the optimal concentrations for reducing mRNA clusterin levels, concentrations of 10, 100, 500, and 1,000 nM were assayed, and a concentration of 100 nM was found to produce maximal reduction in clusterin mRNA levels. At none of the concentrations employed were clusterin levels affected by mismatch control oligonucleotides.

Dose-response curves for chemotherapeutic agents were also produced to determine the appropriate dosages. Thus, breast tumor cells were incubated once daily for 2 days with antisense oligonucleotides at a concentration of 100 nM and then incubated with chemotherapeutic agents for 1 day (at 10^-7 ^and 10^-8 ^M).

### Antibody treatment of cells

Goat antilipoprotein J polyclonal was provided by Chemicon International (Temecula, CA, USA). Taking into account a previous report, concentrations of 20, 30, and 40 μg/mL were assayed [18]. A dose of 30 μg/mL was found to have the maximal effect on breast carcinoma cells. In assays with chemotherapeutic agents, the cells were incubated for 1 day with the anticlusterin antibody and then treated for a further day with the chemotherapeutic agent.

### Determination of cell viability

The cells were distributed at 50,000 cells per well in 12-well plates and treated with oligonucleotides and the anticancer drugs, and floating and adherent cells were recovered. The number of viable cells was determined by trypan blue exclusion analysis.

### TUNEL assay

After the period of incubation, the cells were immediately fixed by adding 37% formaldehyde and incubating them at room temperature for 30 minutes. The fixed cells were then allowed to dry overnight. To score for apoptosis, a TUNEL (terminal deoxynucleotidyl transferase-mediated dUTP-biotin nick end-labeling) technique was used to detect apoptotic cells in breast cancer cell lines using the Roche *in situ *Apoptosis Detection System in accordance with the manufacturer's protocol. The percentage of positively stained cells per total number of cells per high-power field in five random fields was counted and averaged.

### Statistical analysis

One-way analysis of variance was used to compare means. The level of statistical significance was set at a *p *value of less than 0.05, and all statistical calculations were carried out using SPSS.14 software (SPSS Inc., Chicago, IL, USA).

## Results

### Enhanced expression of clusterin after treatments with dexamethasone, tamoxifen, paclitaxel, and doxorubicin

**Figure 1:**
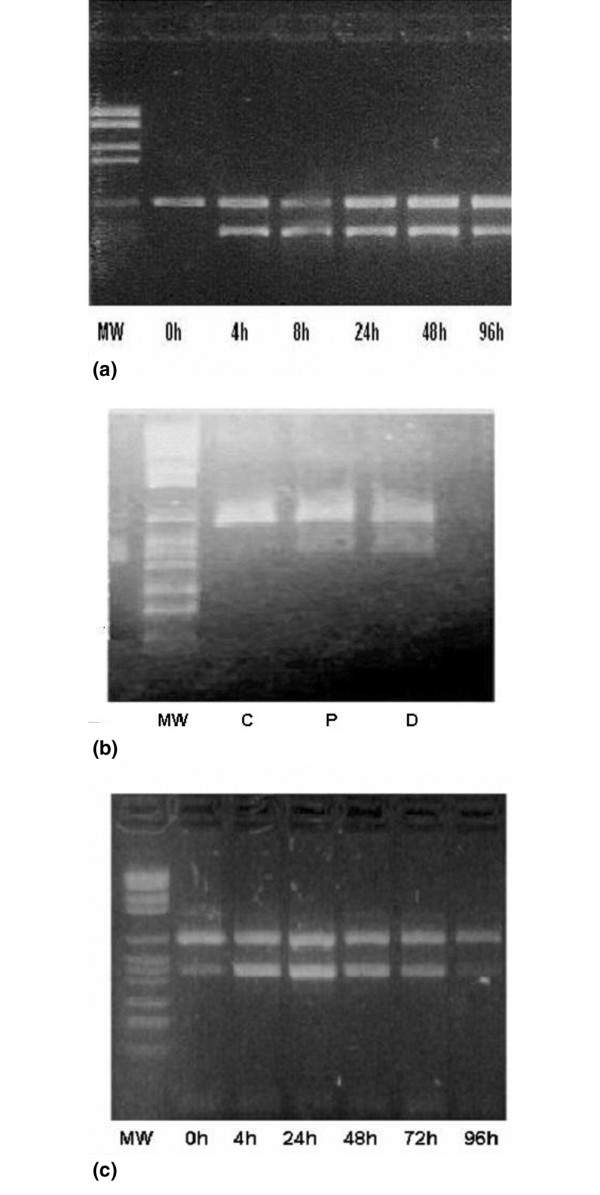
Reverse transcription-polymerase chain reaction analysis showing upregulation of clusterin in MCF-7 tumor cells after treatment with dexamethasone **(a)**, chemotherapeutic agent after 1 day of incubation **(b)**, and tamoxifen **(c)**. C, basal expression; D, doxorubicin; MW, molecular weight; P, paclitaxel.

The MCF-7 cells treated with 1 × 10^-7 ^M dexamethasone (a pharmacologically achievable dose) expressed maximal levels of clusterin by reverse transcription (RT)-PCR as early as 4 hours after induction, and these high levels were maintained even at 4 days after treatment (Figure 1). After this point, the level began to decrease (data not shown). In the MDA-MB-231 cell line, which expresses clusterin at high levels in basal conditions, no significant changes were observed in clusterin expression (data not shown).

RT-PCR analysis was also used to determine the effects of chemotherapeutic agents and tamoxifen on clusterin RNA expression in breast cancer cell lines. As shown in Figure 1, clusterin RNA induction increased in the MCF-7 cell line for both treatments. Time course experiments in breast cancer cells demonstrated that clusterin RNA upregulation peaked as early as 4 to 8 hours after treatment. Again, in the MDA-MB-231 cell line, no significant changes were observed.

### Effects of antisense clusterin on clusterin mRNA and protein expression

**Figure 2:**
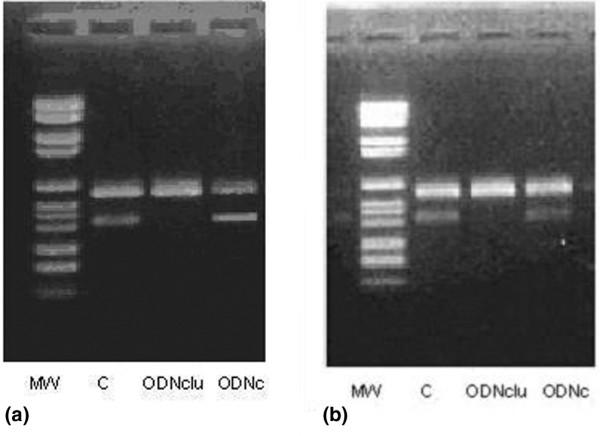
Reverse transcription-polymerase chain reaction analysis showing a strong decrease in clusterin RNA after treatment with antisense oligodeoxynucleotides to clusterin (ODNclu) in the MDA-MB-231 **(a) **and MCF-7 **(b) **cell lines. C, basal expression; MW, molecular weight; ODNc, control oligonucleotide.

RT-PCR analysis was used to determine the effect of treatment with antisense clusterin ODN on clusterin RNA expression in breast carcinoma cells. As shown in Figure 2, treatment of breast tumor cells with 100 or 500 nM antisense clusterin ODN decreased clusterin compared with cells treated with mismatch control ODN. Western blotting confirmed the changes in clusterin protein expression (data not shown).

**Figure 3:**
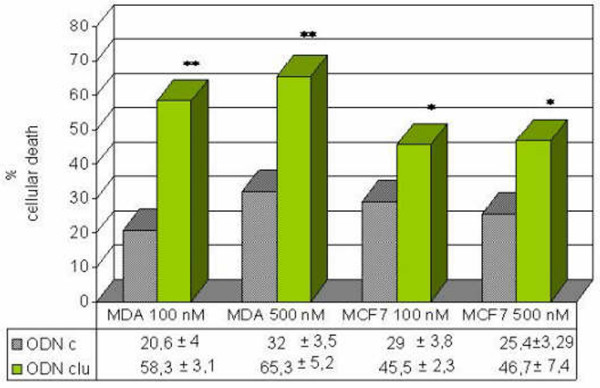
Effects of antisense clusterin oligonucleotide (ODNclu) on cytotoxicity of breast tumor cell lines. Cells were treated daily with 100 or 500 nM antisense ODNclu or clusterin mismatch control ODN (ODNc) for 2 days. Cell viability was determined by trypan blue exclusion test. Each data point represents the mean percentages of at least three independent experiments ± standard error of the mean. *p < 0.05; **p < 0.01.

We determined the effect of antisense clusterin treatment in both cell lines. In the MDA-MB-231 cell line, which expresses high levels of clusterin in basal conditions, antisense clusterin at concentrations of 100 and 500 nM produced a very significant decrease in cell viability. In the MCF-7 cell line, which expresses clusterin at lower levels, this difference was statistically significant (Figure 3). The anticlusterin antibody at a concentration of 30 μg/mL also produced significant results for the percentage of cytotoxicity compared with that produced with a nonimmune immunoglobulin G (IgG) fraction in both cell lines: MDA-MB-231 (28% ± 4.3% with the anticlusterin antibody versus 7% ± 1.5% without the antibody; *p *= 0.002) and MCF-7 (69.6% ± 8.3% with the antibody versus 34% ± 6% without the antibody; *p *= 0.004).

### Antisense oligodeoxynucleotide and antibody to clusterin increase the cytotoxicity of paclitaxel and doxorubicin in MCF-7 and MDA-MB-231

**Figure 4:**
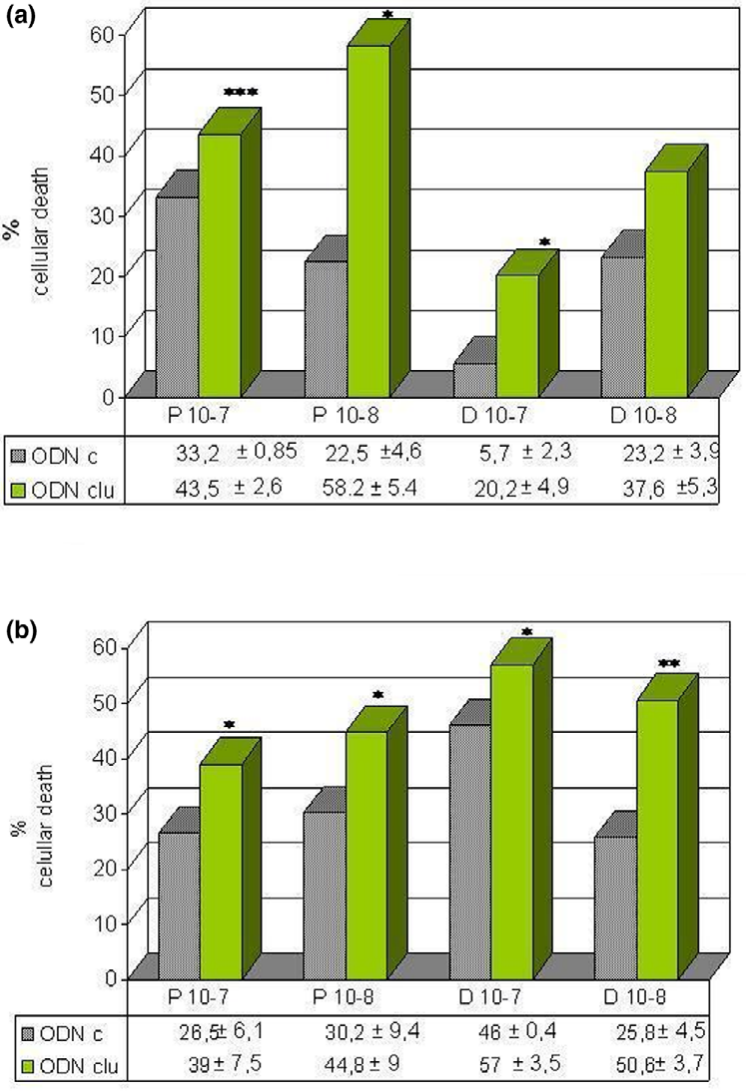
Effects of combined treatment with antisense clusterin oligonucleotide (ODNclu) and paclitaxel or doxorubicin on cytotoxicity of the MDA-MB-231 **(a) **and MCF-7 **(b) **cell lines. Cells were treated daily with 100 nM antisense ODNclu or clusterin mismatch control ODN (ODNc) for 2 days. After ODN treatment, the medium was replaced with medium containing 10^-7 ^and 10^-8 ^M paclitaxel or doxorubicin. Cell viability was determined by trypan blue exclusion test. Each data point represents the mean percentages of at least three independent experiments ± standard error of the mean. D, doxorubicin; P, paclitaxel. *p < 0.05; **p < 0.01; ***p < 0.001.

**Figure 5:**
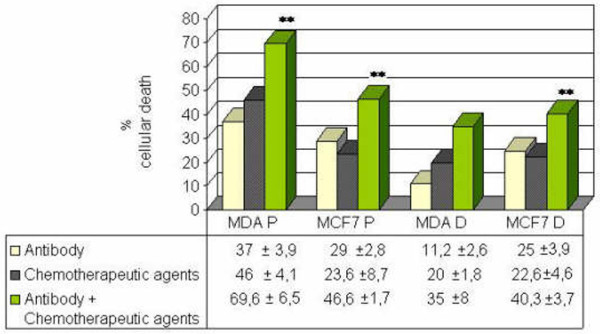
Effects of combined treatment with anticlusterin antibody and paclitaxel or doxorubicin on cytotoxicity of the MDA-MB-231 and MCF-7 cell lines. Cells were treated with the antibody for 1 day (30 μg/mL). After antibody treatment, the medium was replaced in some wells with medium containing 10^-7 ^M paclitaxel or doxorubicin for 1 day. Cell viability was determined by trypan blue exclusion test. Each data point represents the mean percentages of at least three independent experiments ± standard error of the mean. **p < 0.01. Differences were calculated comparing the combined treatment with anticlusterin antibody or chemotherapeutic agent alone. D, doxorubicin; P, paclitaxel.

The combination of antisense clusterin ODN or anticlusterin antibody with the chemotherapeutic drug doxorubicin and paclitaxel produced a significant additive cytotoxic effect in both cell lines (Figures 4 and 5). In addition, TUNEL staining revealed an increased number of apoptotic cells in antisense clusterin ODN-treated MCF-7 cells compared with treatment with mismatch control oligonucleotides plus chemotherapy (61.3% ± 4% versus 37.6% ± 1.4% for paclitaxel, *p *< 0.01, and 79.6% ± 2.6% versus 35% ± 12% for doxorubicin, *p *< 0.05).

### Anticlusterin treatment increases the efficacy of tamoxifen

**Figure 6:**
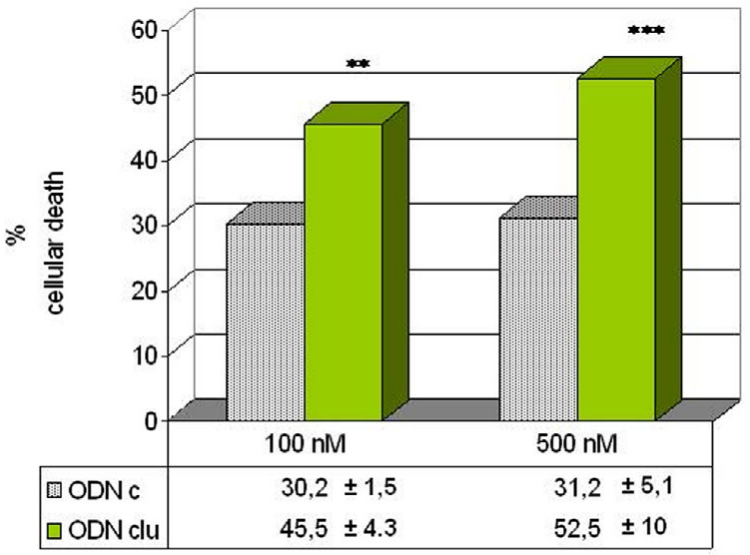
Effects of combined treatment with antisense clusterin oligonucleotide (ODNclu) (at 100 and 500 nM) and tamoxifen (10^-7 ^M) on cytotoxicity of the MCF-7 cell line. The same treatment schedule described in figure 3 was followed. **p < 0.01; ***p < 0.001. ODNc, control oligonucleotide.

MCF-7 cells were treated with tamoxifen (10^-7 ^M) for 1 day and then incubated with antisense oligonucleotides (2 days) or anticlusterin antibody (1 day), after which they were treated with tamoxifen (10^-7 ^M) for 1 day. Again, a significant additive cytotoxic effect was obtained with the combined treatment (Figure 6). Thus, tamoxifen combined with clusterin antibody produced a significant effect on cytotoxicity (57.3% ± 1.7% versus 31.6% ± 5.7%; *p *< 0.01). According to the TUNEL assay, clusterin oligonucleotides also enhanced apoptosis induced by tamoxifen (72.7% ± 2.4% versus 46.7% ± 4.7%; *p *< 0.01) or by tamoxifen plus paclitaxel (80% ± 2.3% versus 59.6% ± 3.1%; *p *< 0.01).

### Dexamethasone pretreatment significantly decreases the efficacy of doxorubicin and paclitaxel

**Figure 7:**
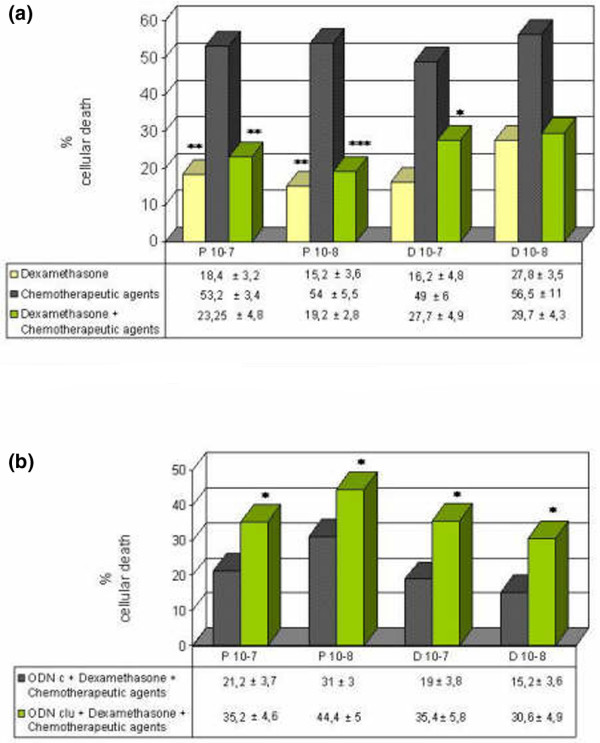
Effects of dexamethasone pretreatment on chemotherapy-induced cytotoxicity. **(a) **Dexamethasone pretreatment (10^-7 ^M) inhibits chemotherapy-induced cytotoxicity in the MCF-7 cell line. **(b) **However, clusterin antisense oligonucleotides (ODNclu) increase chemotherapy-induced cytotoxicity in the presence of dexamethasone. Cells were treated with dexamethasone for 4 hours. After dexamethasone treatment, the medium was replaced in some wells with medium containing 10^-7 ^M paclitaxel or doxorubicin for 1 day. Cell viability was determined by trypan blue exclusion test. Each data point represents the mean percentages of at least three independent experiments ± standard error of the mean. *p < 0.05; **p < 0.01; ***p < 0.001. Differences between treatment with chemotherapeutic agents and treatment with dexamethasone alone or dexamethasone plus chemotherapeutic agents are shown in **(a)**. D, doxorubicin; ODNc, control oligonucleotide; P, paclitaxel.

Dexamethasone pretreatment of breast cancer cell lines is associated with the transcriptional induction of clusterin. As expected, treatment with antisense oligonucleotides to clusterin represses the effect of dexamethasone on clusterin. By the TUNEL method, dexamethasone did not present statistically significant differences in the percentage of apoptosis with respect to control with medium alone after 1 day of incubation in the MCF-7 cell line (10% ± 5% versus 11.6% ± 5.7%, respectively). However, dexamethasone treatment clearly inhibits chemotherapy-induced cytotoxicity (Figure 7a). These findings indicate that the combination of dexamethasone with conventional chemotherapeutic agents may result in antagonistic antitumor effects. However, antisense oligonucleotides to clusterin increase chemotherapy-induced cytotoxicity, even in the presence of glucocorticoid treatment, suggesting a possible role for these proteins in glucocorticoid-mediated survival (Figure 7b). Evaluation of *in situ *apoptosis revealed similar results. Thus, in the treatment with taxol plus dexamethasone, the percentage of apoptosis with control oligonucleotides was 23.3% ± 6.6% versus 46.3% ± 4.6% with antisense oligonucleotides to clusterin.

## Discussion

Although clusterin expression in normal breast epithelial cells is low or absent, it is very high in breast cancer cells during carcinogenesis and metastatic progression [14]. In prostate cancer, the accumulated evidence strongly suggests that clusterin overexpression protects cancer cells from apoptotic cell death induced by several therapies, including hormone and cytotoxic therapy, thereby accelerating progression to androgen independence and conferring chemoresistance [4,8,19]. The combined use of antisense oligonucleotides with cytotoxic chemotherapy has been shown to produce more potent antineoplastic effects [8,19,20].

The present study confirms in the estrogen receptor-positive breast cancer cell line MCF-7 that clusterin levels increase after treatment with tamoxifen and dexamethasone. Previous studies have reported increased clusterin expression after treatment with toremifene [21] and tamoxifen [22]. We also evaluated the effect of cytotoxic treatment on the level of clusterin expression in breast tumor cell lines because this has been shown to be highly upregulated in various tissues undergoing apoptotic cell death [23,24]. As expected, clusterin expression in MCF-7 cells was found to increase considerably after cytotoxic treatment, suggesting that clusterin upregulation is likely to be an adaptative response that mediates chemoresistance. We also investigated the capacity of anticlusterin treatment to sensitize breast carcinoma cells to chemotherapy and searched for drug combinations that produce additive cytotoxicity. Antisense clusterin oligonucleotides or anticlusterin antibodies efficiently inhibited clusterin expression in MCF-7 and MDA-MB-231 cell lines, and this activity was associated with a decrease in cell viability. These findings confirm the cytoprotective function of clusterin in breast carcinoma cells and suggest that there is a role for anticlusterin therapy in the treatment of breast carcinomas that mainly express clusterin protein [11,14].

In addition, we found that in both cell lines, combinations of anticlusterin treatment with chemotherapeutic agents or tamoxifen performed better than the respective single-agent treatments alone. A sequence control oligonucleotide and nonimmune IgG fraction did not increase the effect of chemotherapy, which suggests that the sensitization of cells to apoptosis was due to the specific downregulation of clusterin. A previous report confirmed the chemosensitivity to paclitaxel in breast cancer cell lines [11]. In addition, in other tumors, anticlusterin therapy with antisense oligonucleotides [17,19,25,26] or siRNA [9] has been evaluated as a potential therapeutic agent.

Dexamethasone, a synthetic steroid, is routinely given to women just before they receive chemotherapy with either paclitaxel or doxorubicin, two drugs commonly used to treat breast cancer. Recent findings show that corticoids could protect against chemotherapy-induced apoptosis [12]. Our results show that clusterin expression is increased by dexamethasone treatment. About 4 hours after hormone application, a large increase in clusterin mRNA levels was detectable and was maintained at this high level even at 96 hours after treatment (Figure 1). After this point, the level began to decrease. A similar response by clusterin after treatment with glucocorticoids was obtained on hemangioma *in vitro *[27]. These results, together with the facts that dexamethasone inhibits chemotherapy-induced cytotoxicity and that antisense oligonucleotides to clusterin increase chemotherapy-induced cytotoxicity, clearly show that dexamethasone modulates the expression of clusterin. This effect is in agreement with the known anti-inflammatory properties of dexamethasone in upregulating the expression of other complement inhibitors [28].

## Conclusion

We suggest that glucocorticoids may influence breast cancer behavior via the upregulation of clusterin, which might play a major role in the effects of dexamethasone, protecting breast cancer cells from the effects of both paclitaxel and doxorubicin. Blocking clusterin, on the other hand, reverses the drug's unwanted effects on cancer cell survival. In addition, our studies have firmly established a role for clusterin as a cell survival gene that is increased after tamoxifen therapy and chemotherapy to inhibit tumor cell death. The inhibition of clusterin, using antisense oligonucleotides and antibodies, enhances the cytotoxic effects of chemotherapy agents, including paclitaxel and doxorubicin.

## Abbreviations

IgG = immunoglobulin G; ODN = oligodeoxynucleotide; PCR = polymerase chain reaction; RT-PCR = reverse transcription-polymerase chain reaction; siRNA = small interfering RNA; TUNEL = terminal deoxynucleotidyl transferase-mediated dUTP-biotin nick end-labeling.

## Competing interests

The authors declare that they have no competing interests.

## Authors' contributions

MR conceived of the study, performed the statistical analysis, participated in apoptosis detection techniques, and drafted the manuscript. TT, MJR, and MG-A carried out the cytotoxic assays and participated in RT-PCR analysis and apoptosis detection techniques. AS participated in RT-PCR analysis and the interpretation of the results. MEG critically reviewed the manuscript and contributed to the interpretation of the results. MLH and MM participated in the design of the study and interpretation of the results and helped to draft the manuscript. All authors read and approved the final manuscript.
